# Triethyl­ammonium 2,4-dinitro­phenyl­barbiturate

**DOI:** 10.1107/S1600536809036976

**Published:** 2009-09-26

**Authors:** Doraisamyraja Kalaivani, Rangasamy Malarvizhi

**Affiliations:** a PG and Research Department of Chemistry, Seethalakshmi Ramaswami College, Tiruchirappalli 620 002, Tamil Nadu, India

## Abstract

In the title mol­ecular salt [systematic name: triethylammonium 5-(2,4-dinitrophenyl)-2,6-dioxo-1,2,3,6-tetrahydropyrim­idin-4-olate], C_6_H_16_N^+^·C_10_H_5_N_4_O_7_^−^, the cation and anion are linked by an N—H⋯O hydrogen bond. In the crystal, inversion-related barbiturate rings are centrosymmetrically connected through pairs of N—H⋯O hydrogen bonds, forming *R*_2_^2^(8)*R*_2_^2^(8) ring motifs.

## Related literature

For further information on the anti­convulsant properties of the title compound and general background, see: Kalaivani *et al.* (2008 ▶). For a related structure, see: Craven (1964 ▶). For data on hydrogen-bond motifs in organic crystals, see: Allen *et al.* (1998 ▶).
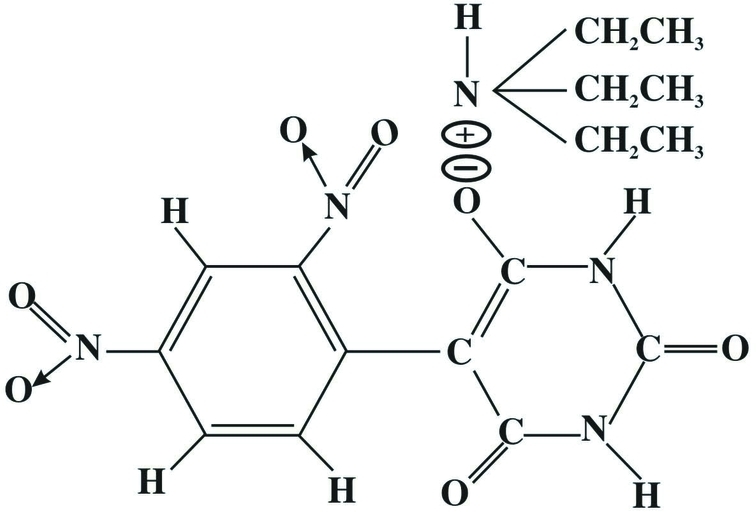


## Experimental

### 

#### Crystal data

C_6_H_16_N^+^·C_10_H_5_N_4_O_7_^−^
*M* *_r_* = 395.38
Monoclinic, 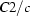

*a* = 29.7900 (8) Å
*b* = 10.4533 (3) Å
*c* = 11.9606 (3) Å
β = 97.903 (1)°
*V* = 3689.20 (17) Å^3^
*Z* = 8
Mo *K*α radiation
μ = 0.11 mm^−1^
*T* = 293 K
0.30 × 0.20 × 0.20 mm

#### Data collection

Bruker Kappa APEXII CCD diffractometer
Absorption correction: multi-scan (*SADABS*; Bruker, 1999 ▶) *T*_min_ = 0.942, *T*_max_ = 0.971
32882 measured reflections
3217 independent reflections
2493 reflections with *I* > 2σ(*I*)
*R*_int_ = 0.036

#### Refinement

*R*[*F* ^2^ > 2σ(*F* ^2^)] = 0.040
*wR*(*F* ^2^) = 0.109
*S* = 1.04
3217 reflections
268 parameters
H atoms treated by a mixture of independent and constrained refinement
Δρ_max_ = 0.46 e Å^−3^
Δρ_min_ = −0.19 e Å^−3^

### 

Data collection: *APEX2* (Bruker, 2004 ▶); cell refinement: *SAINT-Plus* (Bruker, 2004 ▶); data reduction: *SAINT-Plus* and *XPREP* (Bruker, 2004 ▶); program(s) used to solve structure: *SIR92* (Altornare *et al.*, 1993 ▶); program(s) used to refine structure: *SHELXL97* (Sheldrick, 2008 ▶); molecular graphics: *ORTEP-3* (Farrugia, 1997 ▶) and *Mercury* (Macrae *et al.*, 2006 ▶); software used to prepare material for publication: *SHELXL97*.

## Supplementary Material

Crystal structure: contains datablocks global, I. DOI: 10.1107/S1600536809036976/hb5061sup1.cif

Structure factors: contains datablocks I. DOI: 10.1107/S1600536809036976/hb5061Isup2.hkl

Additional supplementary materials:  crystallographic information; 3D view; checkCIF report

## Figures and Tables

**Table 1:** Hydrogen-bond geometry (Å, °)

*D*—H⋯*A*	*D*—H	H⋯*A*	*D*⋯*A*	*D*—H⋯*A*
N1—H1⋯O1^i^	0.84 (2)	2.02 (2)	2.861 (2)	177 (2)
N2—H2⋯O3^ii^	0.82 (2)	2.10 (2)	2.918 (2)	172 (2)
N5—H5⋯O2	0.85 (2)	1.88 (2)	2.730 (2)	172 (2)

Symmetry codes: (i) 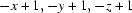; (ii) 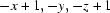.

**Fig. 1.**
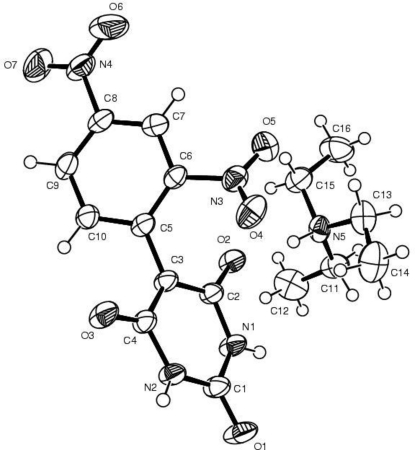
The molecular structure of (I) showing 50% displacement ellipsoids.

**Fig. 2.**
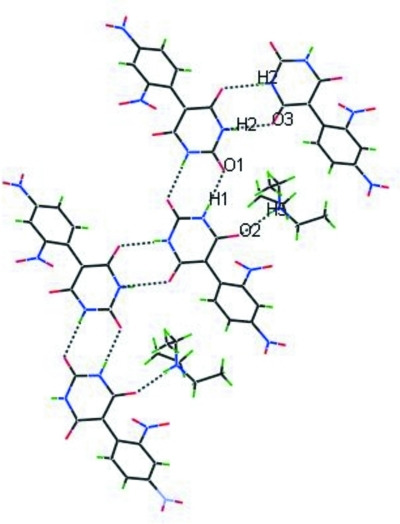
Packing view of (I).

## supplementary crystallographic information

### Comment

We have recently prepared the title compound, (I), which has anticonvulsant activity (Kalaivani *et al.*, 2008).  We now report its crystal structure.

*ORTEP* view of the title compound is shown in Fig. 1. The presence of the leaving group (chlorine atom) *para* with respect to the nitrogroup of the starting molecule (1 – chloro – 2,4 – dinitrobenzene) facilitates the formation of the title compound in the presence of barbituric acid and triethylamine. Absence of chlorine atom, as indicated by the qualitative test on the synthesized barbirutrate has been supported by the crystallographic data. The title molecule is coloured red and it has been attributed to the delocalization of negative charge (Kalaivani *et al.*, 2008) which has also been substantiated by the bond angles and bond lengths of single-crystal X-ray data of 2,4-dinitrophenyl and barbiturate rings. The bond angles and bond lengths of barbiturate residue of the title molecule are compatible with that of barbiturate ion (Craven, 1964), evidencing the delocalization of negative charge in the barbiturate ring.

Presence of double bond (delocalized) between C3 and C5 atoms fixes the configuration of the molecule as depicted in Fig. 1. The N5—H5···O2 hydrogen bond between the asymmetric units is the main driving force for the orientation of the triethylammonium cation (Fig. 2). Two inversion related barbiturate anions interact through a pair of N—H···O=C hydrogen bonds involving N1—H1 atoms and the carbonyl oxygen atom (O1) forming a *R*_2_^2^(8) ring motif. The same ring motif is also due to a pair of N—H···O hydrogen bonds involving N2—H2 atoms and the carbonyl oxygen atom (O3). This motif is one of the 24 most frequently observed bimolecular cyclic hydrogen-bonded motifs in organic crystal structures (Allen *et al.*, 1998). The hydrogen bonds observed in the title molecule are mainly responsible for its stability. The high solubility of the title drug molecule in water (4 g cc^-1^ at 298 K) is due to the positively charged triethylammonium cation and negatively charged 2,4-dinitrophenylbarbiturate anion of the asymmetric unit.

### Experimental

The title compound was prepared as described previously (Kalaivani *et al.*, 2008) and recrystallized from absolute ethanol to yield maroon blocks of (I).

### Refinement

Refinement of F^2^ against ALL reflections. The weighted *R*-factor *wR* and goodness of fit S are based on F^2^, conventional *R*-factors *R* are based on F, with F set to zero for negative F^2^. The threshold expression of F^2^ > 2sigma(F^2^) is used only for calculating *R*-factors(gt) *etc*. and is not relevant to the choice of reflections for refinement. *R*-factors based on F^2^ are statistically about twice as large as those based on F, and *R*-factors based on ALL data will be even larger.

### Crystal data

**Table:**

C_6_H_16_N^+^·C_10_H_5_N_4_O_7_^−^	*F*(000) = 1664
*M**_r_* = 395.38	*D*_x_ = 1.424 Mg m^−^^3^
Monoclinic, *C*2/*c*	Mo *K*α radiation, λ = 0.71073 Å
Hall symbol: -C 2yc	Cell parameters from 8875 reflections
*a* = 29.7900 (8) Å	θ = 2.5–24.5°
*b* = 10.4533 (3) Å	µ = 0.11 mm^−^^1^
*c* = 11.9606 (3) Å	*T* = 293 K
β = 97.903 (1)°	Block, maroon
*V* = 3689.20 (17) Å^3^	0.30 × 0.20 × 0.20 mm
*Z* = 8	

### Data collection

**Table:**

Bruker Kappa APEXII CCD diffractometer	3217 independent reflections
Radiation source: fine-focus sealed tube	2493 reflections with *I* > 2σ(*I*)
graphite	*R*_int_ = 0.036
ω and φ scan	θ_max_ = 24.9°, θ_min_ = 1.4°
Absorption correction: multi-scan (*SADABS*; Bruker, 1999)	*h* = −35→35
*T*_min_ = 0.942, *T*_max_ = 0.971	*k* = −12→12
32882 measured reflections	*l* = −13→14

### Refinement

**Table:**

Refinement on *F*^2^	Primary atom site location: structure-invariant direct methods
Least-squares matrix: full	Secondary atom site location: difference Fourier map
*R*[*F*^2^ > 2σ(*F*^2^)] = 0.040	Hydrogen site location: inferred from neighbouring sites
*wR*(*F*^2^) = 0.109	H atoms treated by a mixture of independent and constrained refinement
*S* = 1.04	*w* = 1/[σ^2^(*F*_o_^2^) + (0.0467*P*)^2^ + 3.5895*P*] where *P* = (*F*_o_^2^ + 2*F*_c_^2^)/3
3217 reflections	(Δ/σ)_max_ < 0.001
268 parameters	Δρ_max_ = 0.46 e Å^−^^3^
0 restraints	Δρ_min_ = −0.19 e Å^−^^3^

### Special details

**Table:**

Geometry. All e.s.d.'s (except the e.s.d. in the dihedral angle between two l.s. planes) are estimated using the full covariance matrix. The cell e.s.d.'s are taken into account individually in the estimation of e.s.d.'s in distances, angles and torsion angles; correlations between e.s.d.'s in cell parameters are only used when they are defined by crystal symmetry. An approximate (isotropic) treatment of cell e.s.d.'s is used for estimating e.s.d.'s involving l.s. planes.
Refinement. Refinement of *F*^2^ against ALL reflections. The weighted *R*-factor *wR* and goodness of fit *S* are based on *F*^2^, conventional *R*-factors *R* are based on *F*, with *F* set to zero for negative *F*^2^. The threshold expression of *F*^2^ > σ(*F*^2^) is used only for calculating *R*-factors(gt) *etc*. and is not relevant to the choice of reflections for refinement. *R*-factors based on *F*^2^ are statistically about twice as large as those based on *F*, and *R*- factors based on ALL data will be even larger.

### Fractional atomic coordinates and isotropic or equivalent isotropic displacement parameters (Å^2^)

**Table:**

	*x*	*y*	*z*	*U*_iso_*/*U*_eq_	
C1	0.49607 (6)	0.30537 (17)	0.47768 (17)	0.0402 (5)	
C2	0.43235 (6)	0.33505 (16)	0.32947 (15)	0.0336 (4)	
C3	0.42904 (6)	0.20117 (16)	0.31524 (15)	0.0339 (4)	
C4	0.45876 (6)	0.11860 (16)	0.38266 (15)	0.0348 (4)	
C5	0.39223 (6)	0.15178 (15)	0.23248 (14)	0.0321 (4)	
C6	0.34717 (6)	0.18925 (16)	0.22995 (14)	0.0317 (4)	
C7	0.31314 (6)	0.15443 (17)	0.14568 (15)	0.0372 (4)	
H7	0.2835	0.1820	0.1463	0.045*	
C8	0.32439 (6)	0.07760 (17)	0.06070 (15)	0.0371 (4)	
C9	0.36810 (7)	0.03485 (18)	0.05876 (16)	0.0408 (5)	
H9	0.3751	−0.0173	0.0006	0.049*	
C10	0.40118 (7)	0.07127 (17)	0.14501 (16)	0.0387 (5)	
H10	0.4305	0.0411	0.1449	0.046*	
C11	0.39539 (7)	0.7557 (2)	0.27509 (19)	0.0498 (5)	
H11A	0.4126	0.7659	0.3495	0.060*	
H11B	0.3779	0.8331	0.2577	0.060*	
C12	0.42740 (9)	0.7390 (3)	0.1905 (2)	0.0700 (7)	
H12A	0.4425	0.6580	0.2022	0.105*	
H12B	0.4495	0.8065	0.1991	0.105*	
H12C	0.4109	0.7419	0.1158	0.105*	
C13	0.33902 (8)	0.6466 (2)	0.37901 (18)	0.0570 (6)	
H13A	0.3268	0.7316	0.3877	0.068*	
H13B	0.3137	0.5877	0.3659	0.068*	
C14	0.36764 (10)	0.6107 (2)	0.48412 (19)	0.0702 (7)	
H14A	0.3803	0.5274	0.4757	0.105*	
H14B	0.3497	0.6092	0.5448	0.105*	
H14C	0.3916	0.6720	0.5006	0.105*	
C15	0.33107 (7)	0.6313 (2)	0.17207 (18)	0.0494 (5)	
H15A	0.3145	0.5521	0.1768	0.059*	
H15B	0.3478	0.6243	0.1083	0.059*	
C16	0.29765 (8)	0.7386 (3)	0.1498 (2)	0.0690 (7)	
H16A	0.2792	0.7424	0.2096	0.104*	
H16B	0.2787	0.7240	0.0793	0.104*	
H16C	0.3136	0.8179	0.1464	0.104*	
N1	0.46707 (5)	0.37959 (15)	0.40826 (14)	0.0416 (4)	
N2	0.49133 (5)	0.17742 (14)	0.46111 (14)	0.0387 (4)	
N3	0.33250 (6)	0.26868 (15)	0.31972 (14)	0.0414 (4)	
N4	0.28841 (7)	0.04383 (17)	−0.03058 (15)	0.0515 (5)	
N5	0.36397 (6)	0.64590 (15)	0.27695 (13)	0.0387 (4)	
O1	0.52439 (5)	0.35119 (13)	0.55104 (13)	0.0575 (4)	
O2	0.40649 (4)	0.41466 (11)	0.27608 (11)	0.0425 (4)	
O3	0.45764 (5)	−0.00016 (12)	0.38115 (12)	0.0496 (4)	
O4	0.35005 (6)	0.25162 (15)	0.41664 (12)	0.0582 (4)	
O5	0.30234 (5)	0.34649 (15)	0.29215 (14)	0.0582 (4)	
O6	0.24947 (6)	0.0672 (2)	−0.01885 (17)	0.0964 (7)	
O7	0.29920 (6)	−0.00325 (19)	−0.11540 (15)	0.0808 (6)	
H1	0.4688 (7)	0.459 (2)	0.4188 (17)	0.046 (6)*	
H2	0.5078 (7)	0.133 (2)	0.5064 (19)	0.050 (6)*	
H5	0.3796 (7)	0.577 (2)	0.2791 (17)	0.044 (6)*	

### Atomic displacement parameters (Å^2^)

**Table:**

	*U*^11^	*U*^22^	*U*^33^	*U*^12^	*U*^13^	*U*^23^
C1	0.0371 (10)	0.0255 (9)	0.0527 (12)	−0.0008 (8)	−0.0122 (9)	−0.0017 (8)
C2	0.0337 (10)	0.0252 (9)	0.0386 (10)	0.0012 (7)	−0.0070 (8)	0.0001 (7)
C3	0.0353 (10)	0.0231 (9)	0.0395 (10)	0.0002 (7)	−0.0087 (8)	−0.0012 (7)
C4	0.0373 (10)	0.0231 (9)	0.0408 (10)	0.0002 (7)	−0.0061 (8)	−0.0025 (7)
C5	0.0393 (10)	0.0194 (8)	0.0344 (10)	−0.0015 (7)	−0.0058 (8)	0.0035 (7)
C6	0.0388 (10)	0.0247 (9)	0.0300 (9)	−0.0034 (7)	−0.0005 (8)	0.0003 (7)
C7	0.0349 (10)	0.0353 (10)	0.0394 (11)	−0.0039 (8)	−0.0016 (8)	0.0028 (8)
C8	0.0443 (11)	0.0295 (9)	0.0335 (10)	−0.0057 (8)	−0.0092 (8)	−0.0003 (8)
C9	0.0537 (12)	0.0284 (9)	0.0382 (10)	0.0004 (9)	−0.0019 (9)	−0.0062 (8)
C10	0.0410 (11)	0.0283 (9)	0.0441 (11)	0.0031 (8)	−0.0044 (9)	−0.0032 (8)
C11	0.0497 (13)	0.0374 (11)	0.0579 (13)	−0.0010 (9)	−0.0077 (10)	0.0042 (10)
C12	0.0664 (16)	0.0652 (16)	0.0797 (18)	−0.0033 (13)	0.0145 (14)	0.0200 (14)
C13	0.0710 (15)	0.0484 (13)	0.0549 (14)	0.0046 (11)	0.0203 (12)	−0.0038 (11)
C14	0.107 (2)	0.0575 (15)	0.0484 (14)	0.0062 (14)	0.0207 (14)	0.0066 (11)
C15	0.0528 (13)	0.0432 (12)	0.0495 (12)	−0.0039 (10)	−0.0029 (10)	−0.0025 (10)
C16	0.0522 (14)	0.0725 (17)	0.0766 (17)	0.0098 (12)	−0.0119 (12)	0.0106 (14)
N1	0.0444 (10)	0.0180 (8)	0.0554 (10)	−0.0014 (7)	−0.0185 (8)	−0.0011 (7)
N2	0.0391 (9)	0.0227 (8)	0.0480 (10)	0.0024 (7)	−0.0161 (8)	0.0015 (7)
N3	0.0436 (10)	0.0370 (9)	0.0435 (10)	−0.0082 (8)	0.0053 (8)	−0.0049 (7)
N4	0.0576 (12)	0.0441 (10)	0.0466 (11)	−0.0041 (9)	−0.0151 (9)	−0.0070 (8)
N5	0.0508 (10)	0.0255 (8)	0.0392 (9)	0.0094 (8)	0.0041 (7)	0.0014 (7)
O1	0.0558 (9)	0.0284 (7)	0.0756 (10)	−0.0022 (6)	−0.0366 (8)	−0.0024 (7)
O2	0.0464 (8)	0.0225 (6)	0.0522 (8)	0.0041 (6)	−0.0162 (6)	0.0014 (6)
O3	0.0594 (9)	0.0205 (7)	0.0600 (9)	0.0023 (6)	−0.0234 (7)	−0.0011 (6)
O4	0.0764 (11)	0.0639 (10)	0.0330 (8)	−0.0074 (8)	0.0031 (7)	−0.0084 (7)
O5	0.0499 (9)	0.0512 (9)	0.0730 (11)	0.0085 (8)	0.0067 (8)	−0.0125 (8)
O6	0.0495 (11)	0.150 (2)	0.0822 (14)	−0.0072 (12)	−0.0177 (10)	−0.0414 (13)
O7	0.0883 (13)	0.0883 (14)	0.0564 (11)	0.0148 (11)	−0.0234 (9)	−0.0350 (10)

### Geometric parameters (Å, °)

**Table:**

C1—O1	1.228 (2)	C12—H12A	0.9600
C1—N2	1.357 (2)	C12—H12B	0.9600
C1—N1	1.357 (2)	C12—H12C	0.9600
C2—O2	1.248 (2)	C13—C14	1.467 (3)
C2—N1	1.381 (2)	C13—N5	1.514 (3)
C2—C3	1.412 (2)	C13—H13A	0.9700
C3—C4	1.408 (2)	C13—H13B	0.9700
C3—C5	1.466 (2)	C14—H14A	0.9600
C4—O3	1.242 (2)	C14—H14B	0.9600
C4—N2	1.396 (2)	C14—H14C	0.9600
C5—C6	1.395 (2)	C15—N5	1.490 (2)
C5—C10	1.397 (3)	C15—C16	1.498 (3)
C6—C7	1.377 (2)	C15—H15A	0.9700
C6—N3	1.471 (2)	C15—H15B	0.9700
C7—C8	1.373 (3)	C16—H16A	0.9600
C7—H7	0.9300	C16—H16B	0.9600
C8—C9	1.380 (3)	C16—H16C	0.9600
C8—N4	1.464 (2)	N1—H1	0.84 (2)
C9—C10	1.379 (3)	N2—H2	0.82 (2)
C9—H9	0.9300	N3—O4	1.217 (2)
C10—H10	0.9300	N3—O5	1.223 (2)
C11—N5	1.483 (3)	N4—O7	1.210 (2)
C11—C12	1.492 (3)	N4—O6	1.212 (2)
C11—H11A	0.9700	N5—H5	0.85 (2)
C11—H11B	0.9700		
			
O1—C1—N2	122.44 (17)	C14—C13—N5	113.4 (2)
O1—C1—N1	122.11 (17)	C14—C13—H13A	108.9
N2—C1—N1	115.45 (16)	N5—C13—H13A	108.9
O2—C2—N1	118.42 (15)	C14—C13—H13B	108.9
O2—C2—C3	124.84 (16)	N5—C13—H13B	108.9
N1—C2—C3	116.74 (15)	H13A—C13—H13B	107.7
C4—C3—C2	120.71 (16)	C13—C14—H14A	109.5
C4—C3—C5	121.58 (15)	C13—C14—H14B	109.5
C2—C3—C5	117.63 (15)	H14A—C14—H14B	109.5
O3—C4—N2	117.69 (16)	C13—C14—H14C	109.5
O3—C4—C3	126.22 (16)	H14A—C14—H14C	109.5
N2—C4—C3	116.04 (15)	H14B—C14—H14C	109.5
C6—C5—C10	115.80 (16)	N5—C15—C16	114.65 (18)
C6—C5—C3	122.95 (16)	N5—C15—H15A	108.6
C10—C5—C3	121.05 (17)	C16—C15—H15A	108.6
C7—C6—C5	123.46 (16)	N5—C15—H15B	108.6
C7—C6—N3	114.89 (16)	C16—C15—H15B	108.6
C5—C6—N3	121.64 (15)	H15A—C15—H15B	107.6
C8—C7—C6	117.81 (17)	C15—C16—H16A	109.5
C8—C7—H7	121.1	C15—C16—H16B	109.5
C6—C7—H7	121.1	H16A—C16—H16B	109.5
C7—C8—C9	121.94 (16)	C15—C16—H16C	109.5
C7—C8—N4	117.70 (18)	H16A—C16—H16C	109.5
C9—C8—N4	120.34 (17)	H16B—C16—H16C	109.5
C10—C9—C8	118.50 (17)	C1—N1—C2	125.39 (16)
C10—C9—H9	120.8	C1—N1—H1	116.9 (14)
C8—C9—H9	120.8	C2—N1—H1	117.4 (14)
C9—C10—C5	122.44 (18)	C1—N2—C4	125.53 (16)
C9—C10—H10	118.8	C1—N2—H2	114.8 (16)
C5—C10—H10	118.8	C4—N2—H2	119.4 (16)
N5—C11—C12	112.55 (18)	O4—N3—O5	123.88 (17)
N5—C11—H11A	109.1	O4—N3—C6	118.73 (16)
C12—C11—H11A	109.1	O5—N3—C6	117.36 (16)
N5—C11—H11B	109.1	O7—N4—O6	123.20 (18)
C12—C11—H11B	109.1	O7—N4—C8	118.14 (19)
H11A—C11—H11B	107.8	O6—N4—C8	118.64 (19)
C11—C12—H12A	109.5	C11—N5—C15	114.20 (16)
C11—C12—H12B	109.5	C11—N5—C13	112.90 (17)
H12A—C12—H12B	109.5	C15—N5—C13	109.90 (17)
C11—C12—H12C	109.5	C11—N5—H5	107.9 (14)
H12A—C12—H12C	109.5	C15—N5—H5	103.4 (14)
H12B—C12—H12C	109.5	C13—N5—H5	107.9 (14)
			
O2—C2—C3—C4	−177.48 (19)	C3—C5—C10—C9	−172.52 (17)
N1—C2—C3—C4	2.8 (3)	O1—C1—N1—C2	−175.1 (2)
O2—C2—C3—C5	−0.7 (3)	N2—C1—N1—C2	4.3 (3)
N1—C2—C3—C5	179.64 (17)	O2—C2—N1—C1	175.63 (19)
C2—C3—C4—O3	176.4 (2)	C3—C2—N1—C1	−4.7 (3)
C5—C3—C4—O3	−0.2 (3)	O1—C1—N2—C4	177.2 (2)
C2—C3—C4—N2	−1.0 (3)	N1—C1—N2—C4	−2.2 (3)
C5—C3—C4—N2	−177.72 (17)	O3—C4—N2—C1	−177.0 (2)
C4—C3—C5—C6	126.7 (2)	C3—C4—N2—C1	0.7 (3)
C2—C3—C5—C6	−50.1 (3)	C7—C6—N3—O4	142.69 (17)
C4—C3—C5—C10	−58.6 (3)	C5—C6—N3—O4	−36.5 (2)
C2—C3—C5—C10	124.63 (19)	C7—C6—N3—O5	−35.6 (2)
C10—C5—C6—C7	−2.2 (3)	C5—C6—N3—O5	145.17 (17)
C3—C5—C6—C7	172.76 (17)	C7—C8—N4—O7	166.62 (19)
C10—C5—C6—N3	176.93 (15)	C9—C8—N4—O7	−12.1 (3)
C3—C5—C6—N3	−8.1 (3)	C7—C8—N4—O6	−11.6 (3)
C5—C6—C7—C8	0.7 (3)	C9—C8—N4—O6	169.7 (2)
N3—C6—C7—C8	−178.44 (16)	C12—C11—N5—C15	−66.8 (2)
C6—C7—C8—C9	0.5 (3)	C12—C11—N5—C13	166.72 (18)
C6—C7—C8—N4	−178.22 (16)	C16—C15—N5—C11	−65.2 (3)
C7—C8—C9—C10	−0.2 (3)	C16—C15—N5—C13	62.8 (2)
N4—C8—C9—C10	178.53 (17)	C14—C13—N5—C11	−73.7 (2)
C8—C9—C10—C5	−1.4 (3)	C14—C13—N5—C15	157.55 (19)
C6—C5—C10—C9	2.5 (3)		

### Hydrogen-bond geometry (Å, °)

**Table:**

*D*—H···*A*	*D*—H	H···*A*	*D*···*A*	*D*—H···*A*
N1—H1···O1^i^	0.84 (2)	2.02 (2)	2.861 (2)	177 (2)
N2—H2···O3^ii^	0.82 (2)	2.10 (2)	2.918 (2)	172 (2)
N5—H5···O2	0.85 (2)	1.88 (2)	2.730 (2)	172 (2)

Symmetry codes: (i) −*x*+1, −*y*+1, −*z*+1; (ii) −*x*+1, −*y*, −*z*+1.
